# Induced growth inhibition, cell cycle arrest and apoptosis in CD133^+^/CD44^+^ prostate cancer stem cells by flavopiridol

**DOI:** 10.3892/ijmm.2014.1930

**Published:** 2014-09-11

**Authors:** BURAK CEM SONER, HUSEYIN AKTUG, EDA ACIKGOZ, FAHRIYE DUZAGAC, UMMU GUVEN, SULE AYLA, CAG CAL, GULPERI OKTEM

**Affiliations:** 1 Necmettin Erbakan University, Meram Faculty of Medicine, Department of Medical Pharmacology, 42100, Konya, Turkey; 2 Ege University Faculty of Medicine, Department of Histology and Embryology, Bornova 35100, Izmir, Turkey; 3 Ege University Health Science Institute, Department of Stem Cell, 35100, Izmir, Turkey; 4 Zeynep Kamil Gynecology and Maternity Training and Research Hospital, Department of Obstetrics and Gynecology, 34668 Istanbul, Turkey; 5 Izmir University of Economics, Faculty of Health Sciences, 35330, Izmir, Turkey

**Keywords:** flavopiridol, prostate cancer, apoptosis, stem cell

## Abstract

Flavopiridol is a flavone that inhibits several cyclin-dependent kinases and exhibits potent growth-inhibitory activity, apoptosis and G_1_-phase arrest in a number of human tumor cell lines. Flavopiridol is currently undergoing investigation in human clinical trials. The present study focused on the effect of flavopiridol in cell proliferation, cell cycle progression and apoptosis in prostate cancer stem cells (CSCs). Therefore, cluster of differentiation 133 (CD133)^+high^/CD44^+high^ prostate CSCs were isolated from the DU145 human prostate cancer cell line. The cells were treated with flavopiridol in a dose- and time-dependent manner to determine the inhibitory effect. Cell viability and proliferation were analyzed and the efficiency of flavopiridol was assessed using the sphere-forming assay. Flavopiridol was applied to monolayer cultures of CD133^high^/CD44^high^ human prostate CSCs at the following final concentrations: 100, 300, 500 and 1000 nM. The cultures were incubated for 24, 48 and 72 h. The half maximal inhibitory concentration (IC_50_) value of the drug was determined as 500 nM for monolayer cells. Dead cells were analyzed prior and subsequent to exposure to increasing flavopiridol doses. Annexin-V and immunofluorescence analyses were performed for the evaluation of apoptotic pathways. According to the results, flavopiridol treatment caused significant growth inhibition at 500 and 1000 nM when compared to the control at 24 h. G_0_/G_1_ analysis showed a statistically significant difference between 100 and 500 nM (P<0.005), 100 and 1000 nM (P<0.001), 300 and 1000 nM (P<0.001), and 500 and 1000 nM (P<0.001). Flavopiridol also significantly influenced the cells in the G_2_/M phase, particularly at high-dose treatments. Flavopiridol induced growth inhibition and apoptosis at the IC_50_ dose (500 nM), resulting in a significant increase in immunofluorescence staining of caspase-3, caspase-8 and p53. In conclusion, the present results indicated that flavopiridol could be a useful therapeutic agent for prostate CSCs by inhibiting tumor growth and malignant progression, and inducing apoptosis.

## Introduction

Flavopiridol (11, NSC649890, L86-8275), [cis-5,7-dihydroxy-2-(2-chlorophenyl)-8-[4-(3-hydroxy-1-methyl)-piperidinyl]-1-benzopyran-4-one] is a semisynthetic flavonoid that is originally isolated from the indigenous Indian plant, *Dysoxylum binectariferum* (1). More specifically, flavopiridol effects tumor cells through cytostatic activity and supports cell cycle arrest and apoptosis. This small molecule is an inhibitor of multiple cyclin-dependent kinases (CDKs), including CDK2, CDK4 and CDK6, which directly compete with adenosine triphosphate at pharmacological doses. This inhibition blocks cell cycle progression and induces G_1_-phase arrest and apoptosis through negative regulation of the phosphoinositide-3 kinase/protein kinase B signaling pathway (2,3). Flavopiridol downregulates bcl-2 mRNA and protein expression (4), and potently interacts with multidrug resistance protein 1 (5). In rhabdoid tumors, the combined treatment of flavopiridol with 4-hydroxy-tamoxifen potentiates their effect and results in apoptosis via induction of caspases 2 and 3. Abrogation of p53 enhances the potency of flavopiridol (6). Furthermore, it exhibits transcriptional suppression activity that downregulates the genes associated with the proliferation pathway (7). Flavopiridol is the first CDK inhibitor to enter clinical trials and a further study supports the relevance of this drug in different organ tumors (8). A phase II consortium study has been conducted with flavopiridol in metastatic renal cancer. The results of this study showed that flavopiridol is not effective and that serious adverse effects, such as vascular thrombotic events and asthenia, have been more frequently observed (9). However, flavopiridol has been shown to sensitize the effect of doxorubicin in small cell lung cancer (SCLC) cells. The study by Budak-Alpdogan *et al* (10) demonstrated that sequential treatment of flavopiridol and doxorubicin induces potent *in vitro* and *in vivo* synergism in retinoblastoma protein (pRb)-negative SCLC cells and should be clinically tested in tumors lacking functional pRb. Despite advances in cancer treatment, therapy is not successful for a number of patients and results in disease recurrence, progression and a decreased overall survival rate. Recent evidence indicates the existence of different cell types in the tumor, and this complexity constitutes a heterogeneous cancer cell population in the tumor mass (11). A minor subpopulation of cancer cells, the cancer stem cells (CSC), are considered to be responsible for tumor initiation and development, metastatic spreading and resistance to radio- and chemotherapy (12). Normal stem cell CSCs fall into asymmetric cell division and this generates one daughter cell that becomes a committed progenitor. As a result of this, hierarchies of actively proliferating, as well as progressively differentiating, cancer cells are formed and this results in the cellular heterogeneity of human cancers (13). Our previous studies demonstrated that when CD133^+^/CD44^+^ prostate CSCs constitute a complex and organized formation, the cellular signaling in the surrounding tissue differ in their behavior (14,15). CSCs, which are reflected as altered expression profiles, and cyclins are significantly upregulated in this differentiation process (16). To the best of our knowledge, there are no previous studies evaluating the growth inhibition of flavopiridol on human prostate CSCs. The current study aimed to investigate the effects of flavopiridol on the viability, sphere formation and apoptosis of CD133^+high^/CD44^+high^ prostate CSCs.

## Materials and methods

### Cell culture conditions and reagents

The DU145 human prostate cancer cell line was supplied by American Type Culture Collection (Manasas, VA, USA) and was grown in monolayer culture in Dulbecco’s modified Eagle’s medium-F12 (DMEM-F12; Biological Industries, Kibbutz Beit-Haemek, Israel) supplemented with 10% heat-inactivated fetal calf serum (Gibco, Invitrogen Life Technologies, Paisley, UK), 100 U/ml penicillin and 100 μg/ml streptomycin (Sigma-Aldrich, St Louis, MO, USA). Cells in semi-confluent flasks were harvested using 0.05% trypsin (Sigma-Aldrich), centrifuged (Nuve NF200; Laboratory and Sterilization Technology, Ankara, Turkey) following the addition of DMEM-F12 for trypsin inactivation, and re-suspended in culture medium. The antibodies used were anti-caspase-3 (1:100 diluted; 3510-100, BioVision, Inc., Milpitas, CA, USA), anti-caspase-8 (1:100 diluted; 250576, Abbiotec, USA), anti-p53 (1:100 diluted; 3036R-100, BioVision, Inc.) and goat anti-rabbit immunoglobulin G-fluorescein isothiocyanate (FITC) (1:100 diluted; sc-2012, Santa Cruz Biotechnology, Inc., Santa Cruz, CA, USA).

### Fluorescence-activated cell sorting (FACS)

For FACS (FACSAria; BD Biosciences, San Jose, CA, USA), the cells were detached using non-enzymatic cell dissociation solution (Sigma-Aldrich) and ~5×10^4^ cells were incubated with an antibody (diluted 1:100 in FACS wash with 0.5% bovine serum albumin; 2 mM NaN_3_ and 5 mM EDTA) for 15 min at 4°C. An isotype and concentration-matched phycoerythrin (PE)-labeled control antibody (Miltenyi Biotec Ltd., Woking, Surrey, UK) was used and the samples were labeled with PE-labeled CD133/1 (clone AC133/1; Miltenyi Biotec Ltd.) and FITC-labeled CD44 (clone G44-26; BD Biosciences). After 3–5 min, the cells were washed with the FACS wash, and subsequently the cells were re-suspended. The cells were organized into a CD133^high^/CD44^high^ subpopulation to become CSCs.

### Analysis of viability and cell death

The investigated drug, flavopiridol, was applied to monolayer cultures of CD133^high^/CD44^high^ human prostate CSCs at the following final concentrations: 100, 300, 500 and 1000 nM, except for the control cells, to which nutrient medium was applied. The cultures were incubated for 24, 48 and 72 h. The half maximal inhibitory concentration (IC_50_) value of the drug was determined as 500 nM for monolayer cells. Dead cells were analyzed prior and subsequent to exposure to increasing flavopiridol doses using the Muse™ Count and Viability kit and the Muse™ Annexin V and Dead Cell Assay kit (Muse™Cell Analyzer; Millipore, Billerica, MA, USA) according to the manufacturer’s instructions.

### Constitution of spheroids and sphere formation assay

For the spheroid cultures, the CD133^high^/CD44^high^ human prostate CSCs were grown as a monolayer and re-suspended with trypsin. The clonogenic potential of the different phenotypic populations was analyzed in a 3D non-adherent culture condition. The liquid overlay technique was used for the constitution of spheroids (16). Briefly, the cells were counted, re-suspended and plated with 1×10^4^ cells per well in a 6-well plate (plates coated with 3% Noble agar) (Difco Laboratories, Inc.; BD Diagnostic Systems, Detroit MI, USA) and incubated at 37°C under 95% air/5% CO_2_. Fresh medium was added every 2–3 days to remove cellular debris and the spheroids that were not well-formed. Two weeks after initiation, the plates were inspected for colony (sphere) growth.

### IC_50_ for flavopiridol on prostate CSC spheroids growth

Flavopiridol was applied to the spheroids to measure the IC_50_ doses at the beginning of the multicellular tumor spheroid formation, incubated at 37°C and protected from light. Flavopiridol plus DMEM was replaced every three days. In the second set of experiments, flavopiridol was added at the IC_50_ dose to day-15 mature spheroids and incubated for 24, 48 and 72 h. The number and diameter of colonies within each well was counted each day under the microscope (Olympus BX-51; Olympus, Hamburg, Germany) and the images were captured for the representative fields.

### Immunofluorescence staining

CD133^+high^/CD44^+high^ prostate CSCs were treated as indicated above and were harvested and fixed in 4% paraformaldehyde for 30 min. Subsequently, the cells were rendered permeable with 0.1% Triton X-100 for 10 min at room temperature, and blocked with phosphate-buffered saline containing 5% bovine serum albumin for 2 h. Following incubation with antibodies against caspase-3, caspase-8, p53 and bcl-2 overnight at 4°C, the cancer cells were treated with FITC-conjugated secondary antibody for 3 h at room temperature. The cells were counterstained with 4′,6-diamidino-2-phenylindole and assessed by a fluorescence microscope equipped with a camera (Olympus BX-51 and the Olympus C-5050 digital test). Statistical analysis was tested by one-way analysis of variance, followed by Tukey’s or Dunett’s post hoc test. P<0.05 was considered to indicate a statistically significant difference.

## Results

### Purity of CD133^high^/CD44^high^-sorted subpopulations and sorting rates

**Figure 1:**
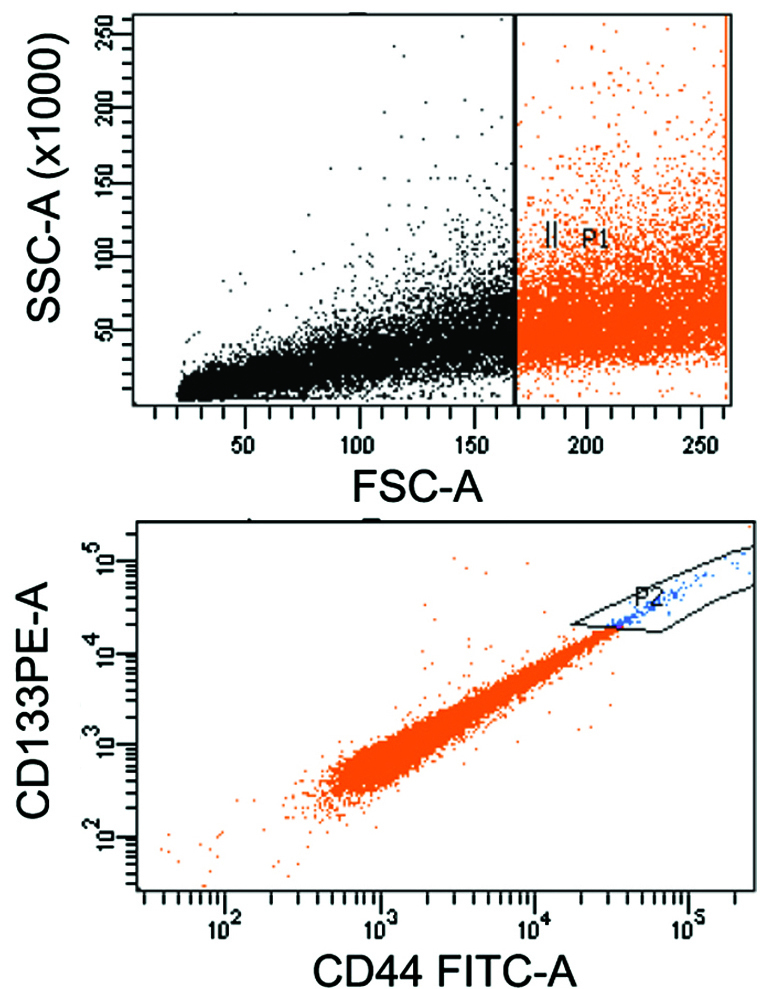
CD133^high^/CD44^high^ prostate cancer stem cells isolated with the FACS-Aria cell sorter. CD133^high^/CD44^high^ populations presented in P2. CD, cluster of differentiation; FACS, fluorescence-activated cell sorting.

DU145 human prostate cancer cells were separated with FACS as the CD133^high^/CD44^high^ population (sorted cells) (Fig. 1). The purity of the CSCs samples were tested with CD133 and CD44 antibodies. The sorting rate analysis and purity of the cells was evaluated sequentially and the rate was 96.7±5.4% for the sorted cells. In order to confirm the flow cytometry analyses, the cells were re-evaluated following sorting and the analyses were repeated after one passage. The results showed that the cell purity following sorting was 85%. Immunofluorescence staining yielded a cell purity of >85% in all the samples (Fig. 1).

### Increasing cytotoxicity of CD133^high^/CD44^high^ prostate CSCs with flavopiridol

**Figure 2:**
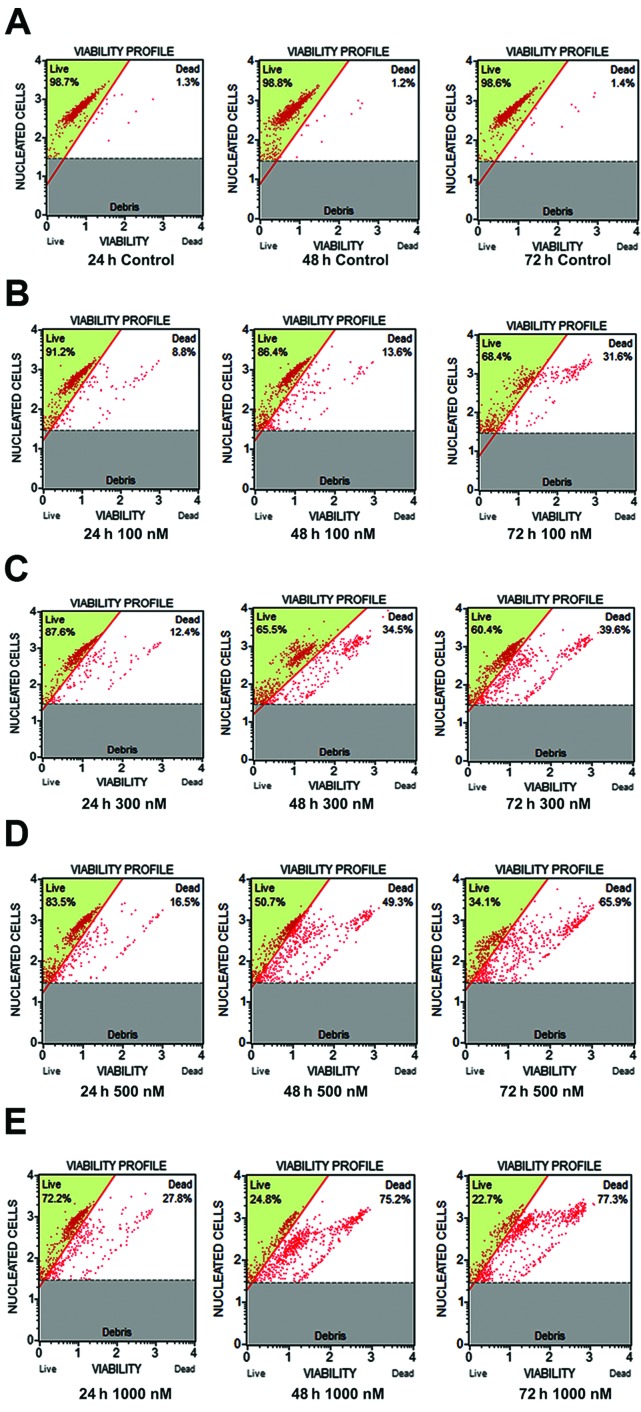
A representative sample of the cell viability profile obtained by treating CD133^+high^/CD44^+high^ prostate cancer stem cells with flavopiridol according to the Muse™ cell analyzer. (A) Control, (B) 100 nM, (C) 300 nM, (D) 500 nM and (E) 1000 nM As shown, flavopiridol efficiently kills CD133^+high^/CD44^+high^ prostate cancer stem cells *in vitro*, but at high concentrations it significantly kills more cells (P<0.001). CD, cluster of differentiation.

**Figure 3:**
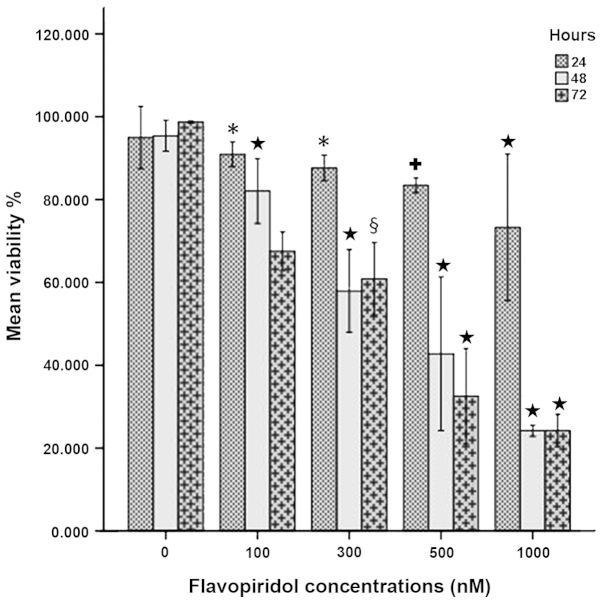
Cell viability following flavopiridol treatment. There was no significant decrease in cell viability at the low flavopiridol doses (100 and 300 nM) for 24 h (^*^P>0.05) compared to the control. There was no statistically significant change between 100 and 300 nM treatment for 72 h (^§^P=0.093). Flavopiridol treatment caused significant growth inhibition in 500 (^+^P=0.018)and 1000 nM (^★^P<0.001) compared to the control at 24 h. Treatment for 48 and 72 h significantly decreased the cell viability of the population at 300, 500 and 1000 nM (^★^P<0.001).

Treated cells were subjected to flavopiridol and are shown in Fig. 2. Flavopiridol reduced the cell viability of CSCs in a dose-dependent manner. According to the data, there were no significant decreases in cell viability at the low doses (100 and 300 nM) of flavopiridol treatment for 24 h (P>0.05) when compared to the control. There was no statistically significant change between 100 nM when compared to 300 nM for 72 h (P=0.093). However, flavopiridol treatment caused significant growth inhibition at 500 (P=0.018) and 1000 nM (P<0.001) when compared to the control at 24 h. Treatment for 48 and 72 h significantly decreased the cell viability of the population at 300, 500 and 1000 nM (P<0.001) (Fig. 3). Therefore, 500 nM was chosen as the optimal dose to be used in the subsequent experiments.

### Cell cycle regulation with high-dose flavopiridol treatment

**Figure 4:**
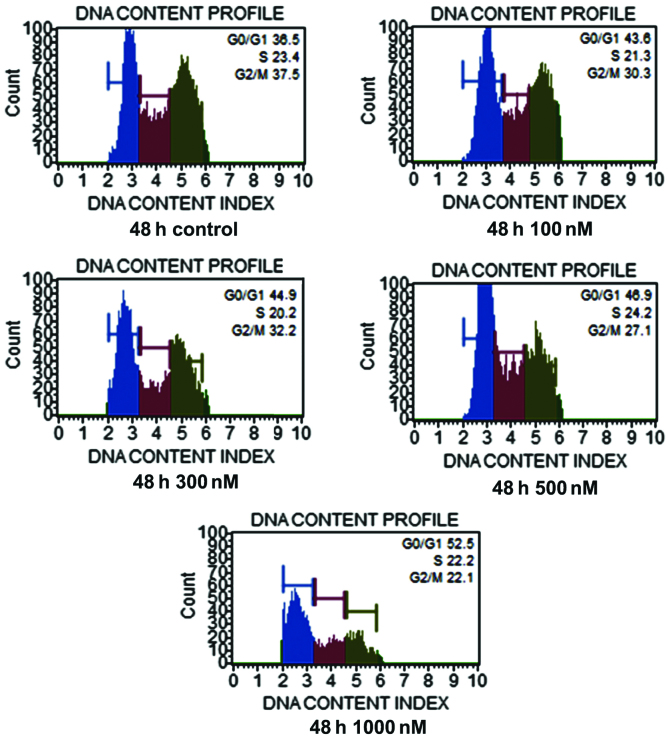
Cell cycle analysis obtained by treating CD133^+high^/CD44^+high^ prostate cancer stem cells with flavopiridol according to the Muse™ cell analyzer. Notably, flavopiridol significantly influenced the cells in the G_0_/G_1_ phase, particularly in the high-dose treatment. CD, cluster of differentiation.

**Figure 5:**
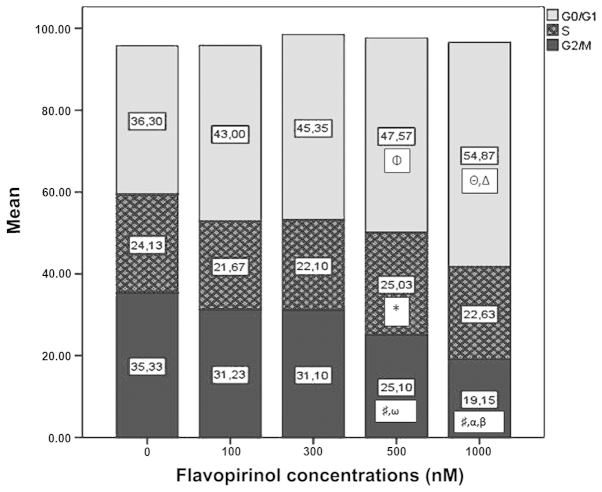
G_0_/G_1_ analysis showing the statistically significant difference between 100 and 500 nM (^Φ^P<0.005), 100 and 1000 nM (^Δ^P<0.001), 300 and 1000 nM (^Δ^P<0.001), 500 and 1000 nM (^Δ^P<0.001) flavopiridol treatment. Flavopiridol affected the S phase of prostate cancer stem cells at the 500 nM dose only compared to 100 nM (^*^P=0.046). For G_2_/M, there was a significant difference between the control and 500 and 1000 nM (^#^P<0.001). Dose-dependent G_2_/M-phase arrest was also observed between 100 and 1000 nM (^α^P<0.002), 300 and 500 nM (^ω^P=0.022), 300 and 1000 nM (^β^P<0.001).

G_0_/G_1_ analysis showed that there were statistically significant changes between 100 and 500 nM (P<0.005), 100 and 1000 nM (P<0.001), 300 and 1000 nM (P<0.001), and 500 and 1000 nM (P<0.001) (Fig. 4). In this phase, CSCs were effectively influenced by 1000 nM treatment and G_0_/G_1_ arrest was observed. Flavopiridol effected the S phase of prostate CSCs at the 500 nM dose only when compared to the other doses (P=0.046). Of note, flavopiridol significantly influenced the cells in the G_2_/M phase, particularly in high-dose treatments. Statistically significant differences were apparent between the control and 500 (P<0.001) and 1000 nM (P<0.001). Dose-dependent G_2_/M-phase arrest was also observed between 100 and 1000 nM (P<0.002), 300 and 500 nM (P=0.022), and 300 and 1000 nM (P<0.001) (Fig. 5).

### Caspase-3, caspase-8 and p53 modulate flavopiridol-associated apoptosis in prostate CSCs

**Figure 6:**
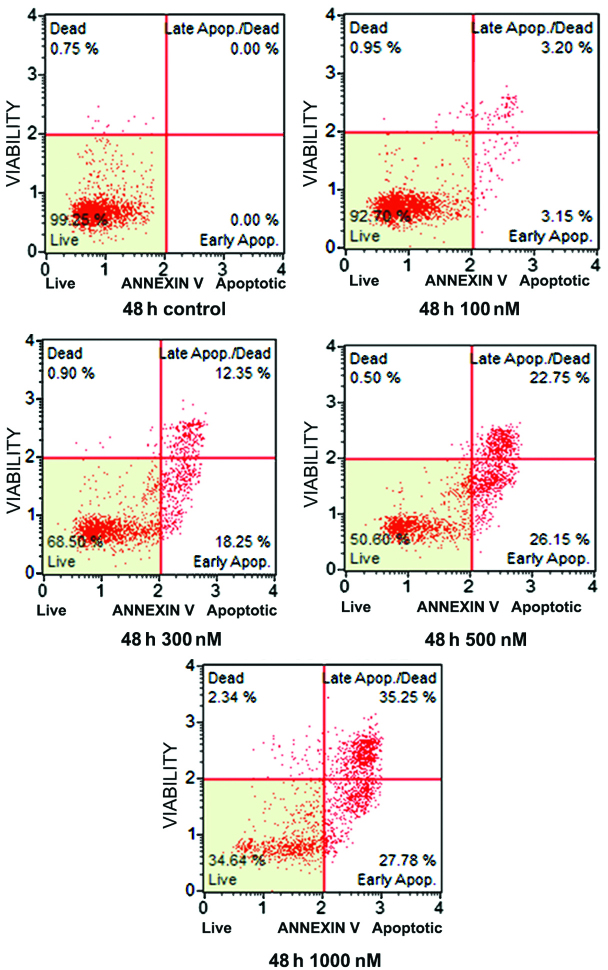
Flavopiridol induced apoptosis in a dose-dependent manner as measured by the Muse™ Annexin V and Dead Cell assay. Flavopiridol was shown to induce apoptotic cell death in the cancer stem cells as mainly early and late apoptosis, which was apparent when the percentage of live cells significantly decreased.

**Figure 7:**
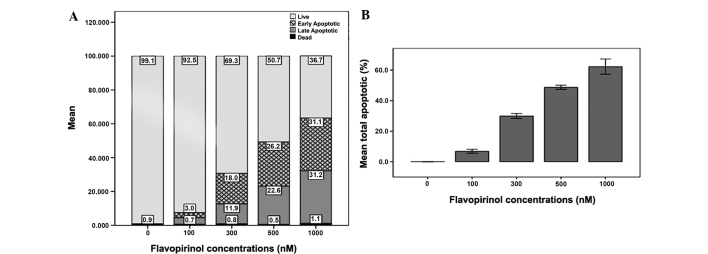
(A) Early and late apoptotic cell numbers increase significant ly (P<0.001) following flavopiridol treatment. (B) Increased flavopiridol treatment induced apoptosis and the most effective concentration was 1000 nM when compared to 100, 300 and 500 nM.

Flavopiridol induced apoptosis in a dose-dependent manner as measured by the Muse™ Annexin V and Dead Cell assay (Fig. 6). Apoptosis was evaluated as early apoptotic cells, late apoptotic cells and dead cells. In this analysis the number of dead cells were significantly decreased, whereas the apoptotic cells increased. Regarding this analysis, it should be noted that flavopiridol induced apoptotic cell death in CSCs mainly as early and late apoptosis, and even the percentage of live cell numbers significantly decreased. Early and late apoptotic cell numbers were increased and were significant different (P<0.001) (Fig. 7A).

The total apoptotic cells were quantified and this demonstrated that flavopiridol induced apoptosis in a dose-dependent manner. Increased flavopiridol treatment induced apoptosis and the maximum effective concentration was 1000 nM when compared to 100, 300 and 500 nM (Fig. 7B).

**Figure 8:**
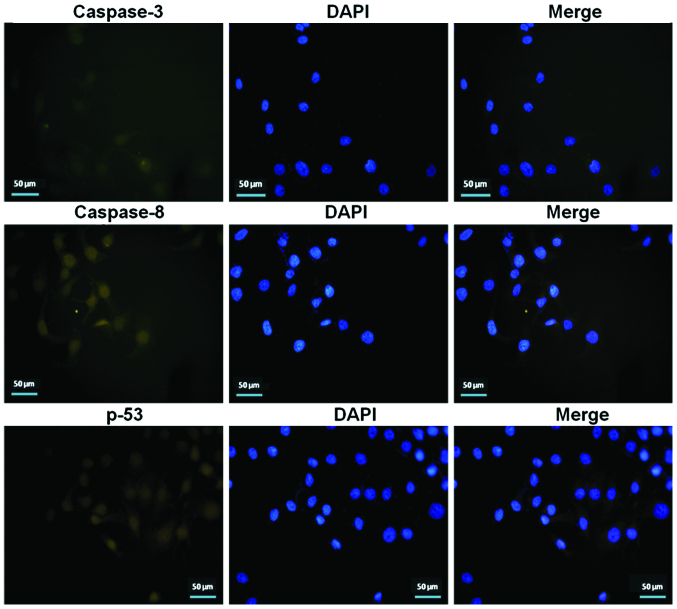
Immunofluorescence analysis showing weak immunoreactivity was observed in pretreated prostate cancer stem cells for caspase-3, caspase-8 and p53.

**Figure 9:**
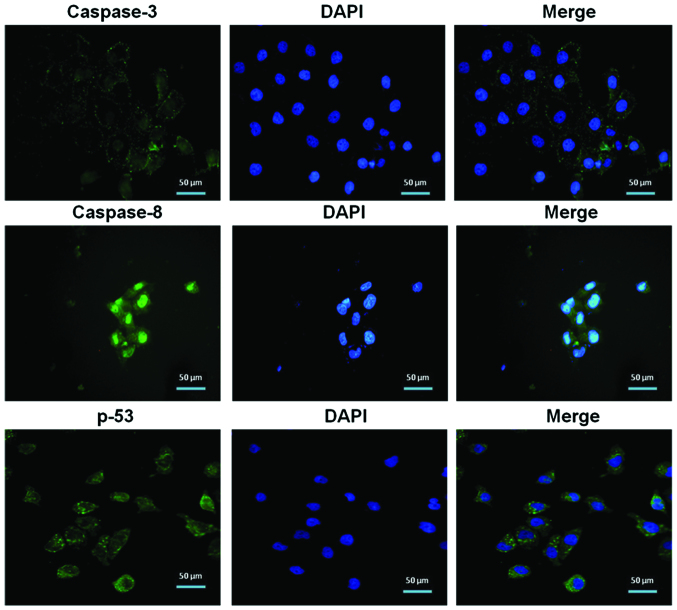
Immunofluorescence staining of caspase-3, caspase-8 and p53 significantly increased following treatment of flavopiridol, which was applied to cells at the IC_50_ dose (500 nM).

Immunofluorescence staining for caspase-3, caspase-8 and p53 supports these results and revealed the apoptotic pathway. Decreased immunoreactivity was observed in pretreated prostate CSCs (Fig. 8). Flavopiridol treatment with an IC_50_ dose (500 nM) resulted in a significant increase in immunofluorescence staining of caspase-3, caspase-8 and p53 (Fig. 9).

### Inhibition of sphere formation with flavopiridol

**Figure 10:**
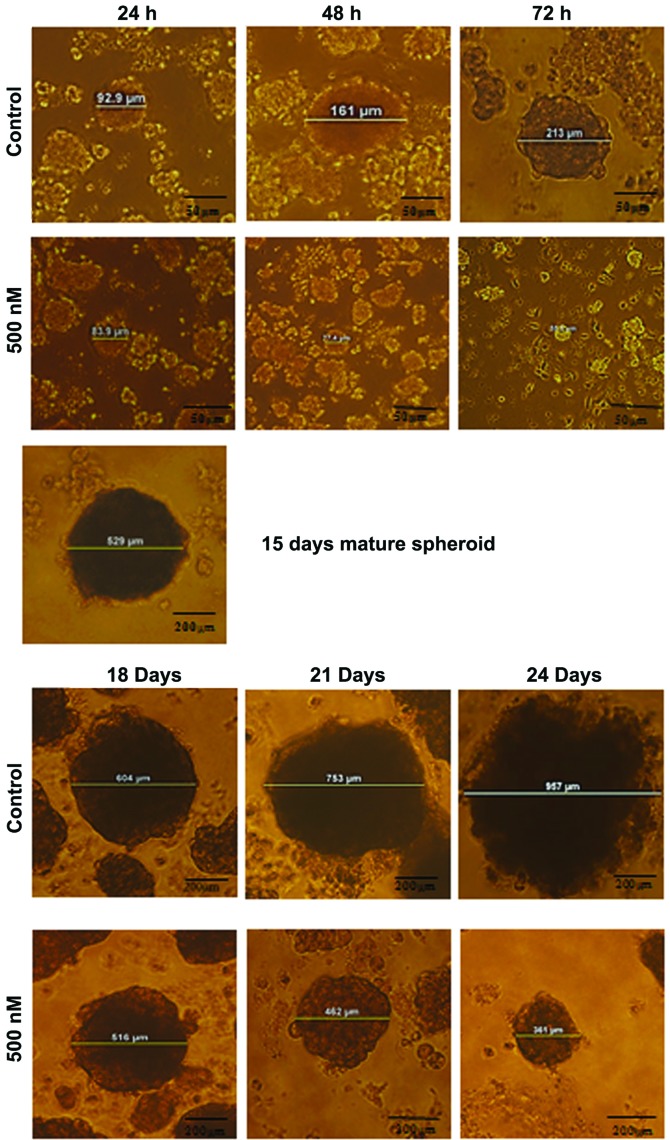
Ability of sphere formation is markedly suppressed in a dose-dependent manner from the beginning of the spheroid constitution and also mature spheroids. Mature spheroid were obtained at 15 day and flavopiridol was added after this day. A significant decrease was observed in the diameter of spheroid formation.

**Figure 11:**
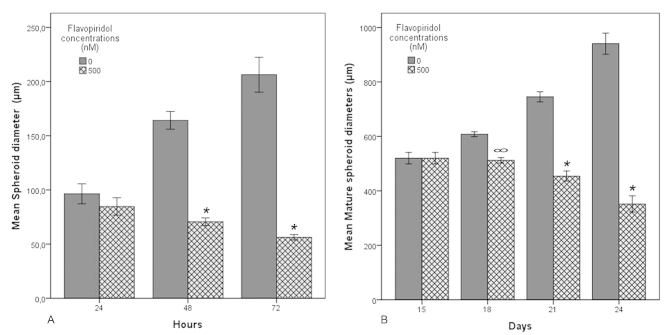
Early spheroid formation inhibition is observed in all the groups (24, 48 and 72 h) following flavopiritol treatment (IC_50_=500 nm) (^*^P<0.001). In the mature spheroids, there was no significant difference between day 15 and 18 flavopiridol-treatment groups (^∞^P=0.931). Excluding this group, significant spheroid formation inhibition was observed in the other groups (P<0.001).

The ability of sphere formation was markedly suppressed in a dose-dependent manner from the beginning of the spheroid constitution and also mature spheroids (Fig. 10). The early and mature spheroids (15 day) were incubated for 24, 48 and 72 h and cells were treated with flavopiridol. The number and diameter of colonies within each well was counted each day under the microscope. Decreased spheroid diameter was observed following flavopiridol treatment. According to statistical analysis, an inhibition of early spheroid formation was observed in all groups (24, 48 and 72 h) at the IC_50_ treatment dose (IC_50_=500 nM) (P<0.001) (Fig. 11). In mature spheroids, there was no significant statistical difference between days 15 and 18 in the flavopiridol treatment group (P=0.931). Besides this group, spheroid formation inhibition was also observed in the other groups and this was significantly different (P<0.001) (Fig. 11). Notably, the downregulation of cell proliferation significantly enhanced the flavopiridol-mediated induction of cell apoptosis and inhibition of sphere formation.

## Discussion

To the best of our knowledge, the present study demonstrated the effect of flavopiridol in prostate CSCs for the first time, and this drug appears to be well-suited as a potential novel agent for the treatment of prostate cancer with supportive clinical trials. The results presented demonstrate that flavopiridol dose-dependently induced growth inhibition and apoptosis in prostate CSCs. Cytotoxic and apoptotic effects of flavopiridol has been shown previously in a bladder cancer cell line (17,18), refractory acute myeloid leukemia (19), rhabdoid tumors (20), germ cell tumors (21) and human cholangiocarcinoma (22). Flavopiridol has been used in a group of relapsed/refractory or *de novo* acute myeloid leukemia patients with a combination regimen known as FLAM (flavopiridol, cytarabine and mitoxantrone) with favorable outcomes and low toxicity. Flavopiridol is one of the novel agents and is also currently under clinical investigation with expectations at the treatment outcomes for chronic lymphocytic leukemia (23,24). The present study is the first, to the best of our knowledge, to evaluate the effects of flavopiridol in CSCs. According to recent studies, cyclin D1 and CDK4/6 have alternate roles in stem-like cell activity and regulation of migration. In addition, these effects are highly dependent of estrogen receptor (ER) expression. Inhibition of cyclin D1 or CDK4/6 increases or decreases migration and stem-like cell activity in ER-negative and -positive breast cancer, respectively (25). Apoptosis has a critical role for cancer treatment, and chemotherapeutic agents are expected to inhibit the growth of cancer cells. Understanding the molecular mechanisms by which flavopiridol may reveal its biological effects on prostate CSCs is important to detect if its efficiency is dependent on alterations of apoptosis-related gene expression and/or induction of apoptosis. The study by Yao *et al* (26) demonstrated that the caspase-3 and Bax proteins were increased significantly in cells treated with flavopiridol, whereas radiation and Bcl-2 protein were significantly decreased. Regarding this, it is believed that flavopiridol promotes Bax and inhibits Bcl-2, thereby promoting caspase-3 and resulting in apoptosis and G_2_/M arrest in the esophageal cancer cell line, Eca109 (26). According to previous studies, flavopiridol down-modulates cyclin D1 and inhibition of its pathway by various mechanisms leads to G_1_ arrest in various cell lines (27–30). Cimica *et al* (6) demonstrated that flavopiridol-induced G_2_ arrest was correlated with downregulation of cyclin B1 and upregulation of p53 and p21. The present study showed that flavopiridol inhibited cell viability and spheroid formation, and induced apoptosis by upregulation of caspase-3, caspase-8 and p53 in CD133^+high^/CD44^+high^ prostrate CSCs. A high dose of flavopiridol also affected CSCs, as the G_2_/M phase was observed as well as G_0_/G_1_ cell cycle arrest in this small subpopulation. Taken together, these findings indicate that flavopiridol could play a potential role in the therapeutic management of prostate cancer.
